# A Clinical Audit of Perioperative Antibiotic Prophylaxis and Intraoperative Monitoring in Orthopaedic Trauma Surgery: An Audit Cycle

**DOI:** 10.7759/cureus.87082

**Published:** 2025-06-30

**Authors:** Arif Khan, Sarah Elmadbouh, Sibthein A Khalid

**Affiliations:** 1 Orthopaedics and Traumatology, Northern Care Alliance, Manchester, GBR; 2 Surgery, NHS England, Cambridge, GBR; 3 Surgery, NHS England, Leeds, GBR

**Keywords:** clincal audit, general trauma surgery, ortho surgery, surg, surgical site infection(ssi)

## Abstract

Introduction

Surgical site infections (SSIs) are a major cause of morbidity following orthopaedic trauma surgeries. Effective perioperative antibiotic prophylaxis, alongside intraoperative blood pressure and temperature monitoring, is essential to reduce infection risk and postoperative complications.

Methods

This retrospective audit was carried out at a National Health Service (NHS) hospital over a three-month period (November 2024 to January 2025). A total of 104 trauma orthopaedic surgical cases were reviewed. The audit assessed adherence to the Local Orthopaedic Surgery Antibiotic Prophylaxis Guidelines and the National Institute for Health and Care Excellence (NICE) standards for intraoperative blood pressure and temperature management.

Results

The audit found that 65.4% (n = 68) of cases were compliant with the recommended intraoperative antibiotic choice. However, 100% (n = 104) of patients received antibiotics within the recommended 60-minute window prior to incision. Postoperative antibiotic compliance was noted in 64.4% (n = 67) of cases, while 24% (n = 25) of patients were prescribed antibiotics for longer than the guideline-recommended duration. Blood pressure monitoring met the National Institute for Health and Care Excellence (NICE) compliance in all cases. In contrast, intraoperative temperature documentation was found in only 35% (n = 36) of cases.

Conclusion

While the timing of antibiotic administration was reliably adhered to, overall compliance with antibiotic selection and postoperative use was suboptimal. Documentation of temperature was particularly poor. A multifaceted quality improvement plan, including electronic patient record (EPR) alerts, mandatory documentation prompts, clinical education, and feedback dashboards, is being implemented. A re-audit is planned in six months to assess the impact of these interventions.

## Introduction

Surgical site infections (SSI) remain a significant complication in trauma orthopaedic surgeries. Optimal perioperative care, particularly around antibiotic prophylaxis, plays a critical role in infection prevention. Local National Health Service (NHS) Trust guidelines and guidelines from the National Institute for Health and Care Excellence (NICE) offer specific recommendations regarding the timing, type, and duration of antibiotic administration, as well as parameters for intraoperative monitoring [1,2]. Haemodynamic stability and normothermia during surgery also play a crucial role in preventing complications and enhancing surgical outcomes. This audit was conducted to assess adherence to these guidelines within the trauma orthopaedic unit at Salford Care Organisation [3].

Although comprehensive, these guidelines are not always consistently followed in clinical practice. Variability in the implementation of perioperative care, such as antibiotic selection, timing, and duration, as well as intraoperative temperature monitoring, may be associated with increased infection rates or inappropriate antibiotic use. Overuse of antibiotics, in particular, contributes to antimicrobial resistance. Therefore, this audit aimed to assess compliance with specific perioperative care guidelines, focusing on antibiotic prophylaxis and intraoperative physiological monitoring, within the trauma and orthopaedic department at a tertiary care hospital.

## Materials and methods

This was a retrospective audit carried out at Salford Care Organisation. The study period spanned from November 2024 to January 2025, reviewing 104 adult patients who underwent orthopaedic trauma surgery. Patients under 16 years of age and those undergoing elective procedures were excluded from the audit.

Data were collected from multiple sources, including electronic patient records (EPRs), Cito-scanned documents, theatre logs, and operative notes. Each case was assessed for compliance with the Northern Care Alliance (NCA) 2023 guidelines [2] regarding perioperative antibiotic prophylaxis and NICE NG180 standards for intraoperative blood pressure and temperature management [1].

Key metrics included adherence to the recommended antibiotic choice and timing, compliance with postoperative antibiotic duration, documentation and maintenance of intraoperative core body temperature between 36.5°C and 37.5°C, and blood pressure management within 20% of baseline.

Data were stratified by procedure type and operating surgeon to identify trends and variability. Results were summarised using descriptive statistics, including the number of compliant cases and corresponding percentages.

## Results

In total, 104 surgical cases were reviewed. Among these, 65.4% (n = 68) received the correct intraoperative antibiotic according to the NCA guidelines. However, all patients (100%, n = 104) received their antibiotics within the recommended 60-minute window prior to incision, as confirmed by the documented administration times in the EPR. Postoperative antibiotic prescribing adhered to guideline-recommended duration in 64.4% (n = 67) of cases. In contrast, 24% (n = 25) received antibiotics for longer than the recommended duration.

Compliance with intraoperative blood pressure monitoring was exemplary, with all 104 patients meeting NICE-defined targets. In contrast, only 35% (n = 36) had documentation of intraoperative core body temperature. Among those with documented temperature readings, normothermia (36.5-37.5°C) was maintained in 100% of cases. Suggesting room for improvement in the consistency of documentation. 

Procedure-specific analysis showed variation in antibiotic compliance across surgery types. Intraoperative compliance was relatively consistent between procedures, while postoperative compliance varied more noticeably, with some procedures showing lower adherence. Antibiotic overuse differed between groups but was less distinctly patterned. Among 27 joint replacement surgeries, intraoperative compliance was 70% (n = 19); postoperative compliance, 40% (n = 11); and antibiotic overuse, 37% (n = 10). Of the three prosthetic joint revision surgeries, only 33.3% (n = 1) were compliant in both intraoperative and postoperative antibiotic use, while 66.7% (n = 2) experienced extended antibiotic use. Internal fixation surgeries showed better adherence, with 64.7% (n = 44) compliant intraoperatively, 75% (n = 51) compliant postoperatively, and 16.2% (n = 11) involving antibiotic overuse (Tables 1-3).

**Table 1:** Intraoperative antibiotic compliance and physiological parameters NCA: Northern Care Alliance Statistical significance was assessed using a one-sample z-test comparing observed compliance against the recommended targets where applicable. While 100% compliance is the clear standard for antibiotic timing and blood pressure maintenance, the expected compliance rate for temperature documentation may realistically be lower due to variable enforcement and recording practices. Therefore, the significant deviation observed for temperature documentation (p < 0.001) likely reflects inconsistent documentation rather than true failure to maintain normothermia

Measure	Compliance (%)	n (total = 104)	Statistical note
Intraop antibiotic choice as per NCA	65.40%	68/104	No test required (single proportion)
Antibiotics given within 60 min	100%	104/104	No variance; test not applicable
BP maintained within 20% of baseline	100%	104/104	No variance; test not applicable
Core body temperature 36.5-37.5°C	35%	36/104	One-sample proportion test: p < 0.001*

**Table 2:** Postoperative antibiotic compliance

Measure	Compliance (%)	n (total = 104)	Statistical note
Postop antibiotic compliance	64.40%	67/104	One-sample z-test vs target (100%): p < 0.001
Overuse (>24 hrs beyond guidelines)	24%	25/104	Descriptive only; no test required

**Table 3:** Procedure-specific trends in antibiotic compliance and overuse Significant differences were observed across procedure types in postop compliance (p = 0.016) and overuse rates (p = 0.048)

Procedure type	Cases (n)	Intraop compliance (%)	Postop compliance (%)	Antibiotic overuse (%)
Joint replacement	27	70%	40%	37%
Prosthetic joint revision	3	33.3%	33.3%	66.7%
Internal fixation	68	64.7%	75%	16.2%
Closed reduction	1	100%	100%	0%
Other orthopaedic	5	60%	80%	40%
p-value (chi-squared)		0.532	0.016	0.048

## Discussion

This audit highlights several important findings. First, adherence to the timing of antibiotic administration was universally achieved, indicating effective preoperative protocols and coordination among the perioperative team. Compliance with intraoperative blood pressure monitoring was similarly robust.

However, antibiotic selection and postoperative duration showed room for improvement. Only 65.4% (n = 68) of cases used guideline-recommended antibiotics intraoperatively, and nearly one-quarter of patients received antibiotics for longer than recommended. These deviations may reflect variability in clinical practice or differences in interpretation of guideline recommendations, rather than clear clinician error. Such patterns were particularly noticeable in joint replacement and revision surgeries, which may involve more complex decision-making due to concerns about infection risk or awaiting microbiology results. Documentation of intraoperative temperature was low, though it is important to note that low documentation does not necessarily indicate poor maintenance of normothermia. Nonetheless, this gap in documentation represents an opportunity for improved recording practices, given evidence linking perioperative hypothermia to adverse outcomes, including coagulopathy and increased infection risk [3].

Previous literature supports the benefits of strict adherence to perioperative antibiotic protocols and temperature monitoring in reducing infection rates and improving patient safety. Uçkay et al. emphasised the role of guideline-based antibiotic use in lowering SSI risk in orthopaedic surgery [4]. Holtom et al. reported that prolonged prophylaxis increases the risk of antimicrobial resistance without additional clinical benefit [5]. The WHO Surgical Safety Checklist has been instrumental in embedding structured safety checks into routine surgical practice [6], while recent global perspectives further reinforce the importance of perioperative standards for infection prevention [7]. Wang and colleagues found that antimicrobial stewardship led by pharmacists significantly improved surgical antibiotic prophylaxis compliance [8].

This audit also identified considerable variability in prescribing practices among individual surgeons, with compliance ranging widely. Such variation may reflect differing clinical case complexities, staffing, or decision-making contexts rather than solely individual habits. These findings underscore the potential value of standardised protocols and enhanced communication to support consistent best practices.

The limitations of this audit include reliance on clinical documentation, which may be incomplete or inconsistent, potentially leading to underestimation of true compliance. Furthermore, inclusion of revision procedures, often requiring extended prophylaxis, may have inflated overuse figures. Future audits may consider excluding such cases or clearly annotating clinical justifications.

## Conclusions

The audit reveals excellent compliance with the timing of antibiotic administration and blood pressure management but identifies significant gaps in the appropriate choice and duration of antibiotic prophylaxis, as well as temperature monitoring. A comprehensive quality improvement plan is being initiated. This includes implementation of EPR-based alerts for antibiotic selection and duration, mandatory intraoperative temperature documentation fields, staff education sessions on guideline updates, and a real-time compliance dashboard for weekly review. A re-audit is scheduled in six months to measure the impact of these interventions and assess ongoing improvement.
